# Effects of Baseline Problematic Alcohol and Drug Use on Internet-Based Cognitive Behavioral Therapy Outcomes for Depression, Panic Disorder and Social Anxiety Disorder

**DOI:** 10.1371/journal.pone.0104615

**Published:** 2014-08-14

**Authors:** Mikael Gajecki, Anne H. Berman, Kristina Sinadinovic, Claes Andersson, Brjánn Ljótsson, Erik Hedman, Christian Rück, Nils Lindefors

**Affiliations:** 1 Center for Psychiatric Research, Department of Clinical Neuroscience, Karolinska Institutet, Stockholm, Sweden; 2 Stockholm Center for Dependency Disorders, Stockholm, Sweden; 3 Health and Society, Department of Criminology, Malmö University, Malmö, Sweden; 4 Department of Clinical Sciences, Division of Psychiatry, Lund University, Lund, Sweden; 5 Department of Clinical Neuroscience, Division of Psychology, Karolinska Institutet, Stockholm, Sweden; 6 Department of Clinical Neuroscience, Division of Psychiatry, Karolinska Institutet, Stockholm, Sweden; Medical University of Vienna, Austria

## Abstract

**Purpose:**

Patients’ problematic substance use prevalence and effects were explored in relation to internet-based cognitive behavioral therapy (ICBT) outcomes for depression, panic disorder and social anxiety disorder.

**Methods:**

At baseline and treatment conclusion, 1601 ICBT patients were assessed with self-rated measures for alcohol and drug use (AUDIT/DUDIT), depressive symptoms (MADRS-S), panic disorder symptoms (PDSS-SR) and social anxiety symptoms (LSAS-SR).

**Results:**

Problematic substance use (AUDIT ≥8 for men, ≥6 for women; DUDIT ≥1) occurred among 32.4% of the patients; 24.1% only alcohol, 4.6% only drugs, and 3.7% combined alcohol and drug use. Hazardous alcohol use and probable alcohol dependence negatively affected panic disorder outcomes, and hazardous drug use led to worse social anxiety outcomes. Depression outcomes were not affected by substance use. Treatment adherence was negatively affected by problematic drug use among men and 25–34 year olds; combined substance use negatively affected adherence for women and 35–64 year olds.

**Conclusion:**

Problematic substance use does not preclude ICBT treatment but can worsen outcomes, particularly problematic alcohol use for panic disorder patients and hazardous drug use for social anxiety patients. ICBT clinicians should exercise particular caution when treating men and younger patients with problematic drug use, and women or older patients with combined substance use.

## Introduction

Considerable evidence indicates an association between psychiatric disorders and problematic substance use. Individuals diagnosed with a psychiatric disorder run double the risk of being diagnosed with an alcohol use disorder and four times the risk of being diagnosed with a drug use disorder, compared to those without a psychiatric disorder [1]. In a US national survey, the 12-month prevalence level of a diagnosed alcohol use disorder among individuals with a mood disorder was 17.3% and 6.9% for a drug use disorder. Among individuals with an anxiety disorder, 12-month prevalences were 13.0% and 4.6% respectively. In comparison, prevalences in the general population were lower – 8.5% for diagnosed alcohol and 2% for drug use disorders. Among those seeking treatment for major depressive disorder, panic disorder with agoraphobia, panic disorder without agoraphobia and social anxiety disorder the 12-month prevalences for an alcohol use disorder were 16.8%, 15.4%, 13.7% and 16.0% respectively. The corresponding numbers for a drug use disorder were 7.5%, 9.7%, 5.1% and 8.2% [2].

In Sweden, about one-fourth of non-psychotic patients at psychiatric outpatient clinics have indicated problematic alcohol use [3], and almost 10% of psychotic patients have problematic drug use [4], higher levels than among the general population, where 21.1% show problematic alcohol use, and 2.8% indicate problematic drug use [5]. The causes of increased substance use among patients with psychiatric disorders are not well studied, but one explanation could be self-medication [6]–[8]. Accordingly, studies have shown that development of common psychiatric disorders such as social anxiety disorder typically precedes the onset of substance abuse [9]–[11]. Initial substance use can conversely induce psychiatric disorders [12]–[14].

Surprisingly, little research seems to have been conducted about the effects of problematic substance use on psychotherapeutic treatment outcomes. A search for studies specifically examining how substance use affects the outcome of psychotherapeutic treatment for depression returned no results. In the area of anxiety disorders, a naturalistic 12-year study showed that concurrent alcohol or other SUDs did not affect social anxiety disorder and panic disorder outcomes [15]. In another study, pre-treatment problematic alcohol use levels did not predict treatment outcomes for social anxiety disorder or panic disorder; however, lower pre-treatment drinking levels led to greater improvement in social interaction anxiety [16]. A systematic review of predictors for dropout and treatment outcomes following CBT for social anxiety disorder examined alcohol and general substance use as predictors for treatment outcomes without finding any association [17].

A recent development in the psychiatric treatment of mental disorders is internet-based cognitive behavioral therapy (ICBT). ICBT has shown effect sizes similar to traditional therapy (*d* = 0.91; 95% confidence interval (CI) 0.68∼1.14) for affective as well as anxiety disorders of mild or moderate severity [18], [19]. Although research on treatment outcomes for internet-delivered psychotherapy for various diagnostic categories has increased exponentially over the past few years [20]–[22], a literature search using the terms “online therapy, e-therapy, alcohol, substance use, alcoholism, drug abuse, drug usage and effect” yielded no research about SUD effects on internet-based psychotherapy treatment outcomes. The little published research available from disparate studies suggests that SUDs do not seem to affect psychiatric treatment outcomes, running counter to standard psychiatric recommendations for integrated treatment for concurrent psychiatric and SUDs, at least for severe mental illness [23]. Furthermore, empirical evidence concerning the prevalence of SUD among patients seeking treatment with ICBT for depression, panic disorder and social anxiety disorder is scarce.

To the best of our knowledge, this is the first study investigating the effects of problematic substance use on ICBT outcomes. We first explored the prevalence of problematic alcohol and drug use among patients treated for depression, panic disorder and social anxiety disorder at an ICBT unit within ordinary psychiatric care in Sweden and, secondly, assessed the effects of problematic substance use on therapy outcomes for these patients.

## Subjects, Materials and Methods

### Ethics statement

All data analyzed in this study came from existing patient registries. After intake, ICBT patients were sent written information that their data might be used in future research regarding treatment effectiveness. The information stated explicitly that the patient could ask for their individual data to be withdrawn from the patient registry. No informed consent form was signed by patients at this time. The study was granted ethical approval by the Stockholm Regional Ethics Review Board, ref nr 2011/1029-31/5, August 18, 2011. The Board thus considered the patient information procedure ethically adequate for this study.

### Setting

The ICBT clinic at Psychiatry Southwest in Stockholm-Huddinge (www.internetpsykiatri.se) specializes in the development and delivery of ICBT within regular health and psychiatric care. Analyses have shown large within-group effect sizes for patients treated for major depressive disorder [24] panic disorder [25], and social anxiety disorder (El Alaoui, S., personal communication 22 october 2013) and maintenance of improvements 6 months after treatment. Baseline screening data on substance use are collected for all patients but the unit has not offered any internet-based treatment specifically for problematic substance use. Treatment at the ICBT unit includes self-assessment and 10–15 diagnosis-specific psycho-educational treatment modules, via a web-based communication platform with online therapist support.

### Recruitment and treatment procedure

Patients are referred for treatment at the ICBT unit by a general practitioner or other physician, or self-referred. They complete web-based screening with diagnosis-specific screening instruments (see under Measures below) and are offered an intake appointment with a resident or specialist psychiatrist for further clinical assessment and diagnosis with the Mini-International Neuropsychiatric Interview (MINI) [26]. All individuals who met with an intake psychiatrist between October 30, 2007 and June 16, 2010, and who went on to initiate treatment, were included in this study. Patients included either had an ongoing unipolar depression, panic disorder (with or without agoraphobia) or social anxiety disorder. At the intake appointment, a joint decision is made by the assessing psychiatrist and the patient on whether or not to begin ICBT treatment. In cases of comorbidity the psychiatrist and the patient jointly decide on which of the offered treatment programs is most suitable for the patient at the time. Patients are informed that participating in other concurrent psychotherapeutic treatment will make them ineligible to receive ICBT. Also, if patients are on medication, their medication needs to be stabilized for one month prior to initiating ICBT. Patients are referred elsewhere if they fulfill any of the following conditions rendering them unable to benefit adequately from the treatment: insufficient knowledge of Swedish, suffering from psychosis or bipolar disorder, substance use so severe it interferes with their ability to participate, or suicidal ideation to a degree that makes other, alternative treatment, more suitable. Approximately 40% of referred patients did not initiate ICBT treatment.

All patients participating in ICBT treatment were given access to an ICBT program with therapist contact via an online asynchronous communication system for a period of 12–15 weeks. The therapists were all clinical psychologists. Patients were instructed to work with one module per week and to contact the therapist at least once every week. Patients could contact their therapist at any time and expect a reply within 48 hours on weekdays. The depression and panic disorder treatments comprised ten modules spanning over 12 weeks. The social anxiety disorder treatment was initially given in 15 modules over 15 weeks, but this was changed after the first year in regular care. The reason for this was that it is clinically more practical to administer a program over 12 weeks in order to avoid longer vacation periods in Sweden when patients are likely to be away (the summer months and winter holidays). In addition, in the original social anxiety disorder treatment, the final three modules were fairly light in content and it was possible to easily compress these into fewer modules with an expected maintained effect. The final format, with 12 modules over 12 weeks, is the one currently used. Moving on to the next treatment module was contingent on completing exercises and answering module-specific questions. Access to therapist support was terminated after 12/15 weeks but access to the text modules was open for additional six months. The treatment context has been described in more detail elsewhere [24], [25].

### Addressing suicidal ideation

All patients complete the Montgomery Åsberg Depression Rating Scale – Self-rated (MADRS-S) weekly. An algorithm alerts the internet therapist, if the patient scores 4 or above on the suicidal ideation item (question 9) and the patient is contacted for in-person assessment. An automated alert is also sent out to the therapist if the patient stops interacting with the system for a period of seven days. The psychologist has access to the patient’s phone number, and calls the patient if s/he stops communicating with the platform. The psychologist can also refer the patient for an emergency psychiatric appointment if deemed necessary. For acute circumstances, every patient has also provided the contact details of a close relative.

### Study population

This study used retrospective data collected from ICBT patients in regular psychiatric care, and included 1601 patients screened for problematic alcohol and drug use, treated for depression (n = 770), panic disorder (n = 483), or social anxiety disorder (n = 347; 170 were offered 15 treatment modules and 177 were offered 12 modules). Twenty patients were excluded from analysis, one due to incorrect information on the number of treatment modules completed and 19 for whom screening data on the Alcohol Use Disorders Identification Test (AUDIT) and the Drug Use Disorders Identification Test (DUDIT) were missing, leaving a total population of 1581.

### Baseline measures

The AUDIT [27] in Swedish translation [28], [29], with well-established reliability and validity, was used to measure alcohol consumption and alcohol-related problems (total score range 0–40). In this study the term problematic alcohol use includes hazardous use (6–15 points for women [28] and 8–15 for men), harmful use (16–19) and probable dependence (20–40) [30]. The DUDIT, also with proven reliability and validity, measured illicit use of drugs and drug-related problems (total score range 0–44). In this study, problematic drug use includes hazardous use (1 for women and 1–5 for men), harmful use (2–24 for women and 6–24 for men) as well as probable dependence (25–44) [29], [31]. The GAF (Global Assessment of Functioning) [32], with demonstrated adequate validity and reliability [33], was used by the intake psychiatrist to rate patients’ functional status (total score range 0–100).

### Continuous measures

MADRS-S (Montgomery Åsberg Depression Rating Scale – Self-rated) [34] was used to measure symptoms and severity of depression. MADRS-S is the self-rating version of MADRS [35], with a total score range of 0–54 [36] and good psychometric properties [37].

PDSS-SR (Panic Disorders Severity Scale – Self-Rating version) [38] was used to measure symptoms of panic disorder. The PDSS-SR is the self-rating version of the PDSS [39] and has a score range of 0–28 [40].

LSAS-SR (Liebowitz Social Anxiety Scale – Self-Rating version) [41] was used to measure social anxiety symptoms and severity. LSAS-SR is the self-rating version of LSAS [42] with a total score range of 0–144 [43]).

All patients completed the MADRS-S at baseline and up to once a week during treatment to measure depression severity. In parallel and at the same intervals, patients treated for panic disorder also completed the PDSS-SR and patients treated for social anxiety disorder completed the LSAS-SR.

### Imputation of missing values and statistical analyses

Missing values at baseline for MADRS-S, PDSS-SR and LSAS-SR were imputed such that missing values were replaced by the first observation carried backward. Post-treatment missing values were replaced by the last prior observation carried forward.

Descriptive statistics were used for participant characteristics and prevalence data. The number of treatment modules completed by gender, age, diagnosis and problematic substance use were evaluated with ANOVA tests. To examine pairwise differences between groups in the analyses of variance, the Tukey-Kramer post-hoc test was used, taking into consideration unequal sample sizes, and controlling for mass significance [44]. Differences in therapy outcome by gender, age, diagnosis and levels of problematic substance use were tested using analyses of variance (ANOVA) and analyses of covariance (ANCOVA).

For purposes of comparison, the number of treatment modules for the social anxiety disorder treatment groups were adjusted to fit a scale of 0–10, and this adjusted number was used in all reported means and analyses.

To simplify interpretation of therapy outcomes over time, within-group effect sizes were calculated as Cohen’s *d* by dividing the mean difference between pre- and post-measures by their pooled standard deviation. Comparisons between effect sizes were based on visual inspection. Interactive effects between treatment outcome and number of modules completed, GAF-scores, gender, age and diagnostic categories were analyzed using analysis of covariance (ANCOVA). All ANCOVA analyses used a reference group of individuals without problematic substance use, who were in the same category (e.g., gender or age). The IBM SPSS Statistical Package version 20 (IBM Corp, Armonk, NY, USA) for MacOS was used for all analyses.

## Results

### Participant characteristics

Table 1 shows participant characteristics by diagnosis for gender, mean age, mean GAF score and number of completed treatment modules, with specified levels of problematic substance use.

**Table 1:** ICBT patient population characteristics by gender, age, global assessment of functioning (GAF) ratings, number of modules completed, and mutually exclusive sub-categories of non-problematic and problematic substance use.

		All	Non-problematicUse	Alcohol Use Categories			Drug Use Categories			Drug Use Categories+Problematic Alcohol Use		
				Hazardous	Harmful	ProbableDependence	Hazardous	Harmful	ProbableDependence	Hazardous	Harmful	ProbableDependence
Total	N (%)	1581(100)	1069(67.6)	321(20.3)	40(2.5)	20(1.3)	44(2.8)	28(1.8)	0(0.0)	23(1.5)	34(2.2)	2(0.001)
	Women (%)	998(63.1)	709(66.3)	207(64.5)	16(40.0)	2(10.0)	11(25.0)	24(85.7)	0.0(0.0)	2(8.7)	27(79.4)	0.0(0.0)
	Mean age (sd)	36.1(11.6)	37(11.6)	34.6(11.4)	33.7(12.0)	35.6(10.0)	35.4(13.6)	35.7(11.3)	-	31(11.0)	29.1(7.8)	24(2.8)
	Mean InitialGAF^a^ (sd)	62.2(8.0)	62.3(8.0)	62.4(8.8)	63.2(6.3)	59.8(6.2)	61.1(5.7)	59.9(6.8)	-	61.7(6.3)	61.8(8.2)	60(0.0)
	Mean CompletedModules (sd)	6.9(3.1)	7.2(3.0)	6.8(3.1)	6.2(3.0)	5.1(3.4)	5.7(3.3)	6.5(3.6)	-	5.3(3.4)	5.8(3.1)	2.7(0.9)
Depression	N (%)	762(100)	508(66.7)	160(21.0)	24(3.1)	11(1.4)	14(1.8)	15(2.0)	0(0.0)	10(1.3)	20(2.6)	0(0.0)
	Women (%)	505(66.3)	358(70.5)	104(65.0)	8(33.3)	1(9.1)	6(42.9)	11(73.3)	0.0(0.0)	1(10.0)	16(80.0)	0.0(0.0)
	Mean age (sd)	38.4(12.1)	39.1(12.0)	37.2(12.1)	36.4(13.1)	38.2(9.6)	44.1(13.8)	37.1(11.1)	-	32.2(11.8)	31.6(8.4)	-
	Mean InitialGAF^a^ (sd)	62.7(7.1)	62.6(7.2)	63.8(6.8)	65.0(6.0)	56.9(5.9)	61.6(6.2)	59.6(6.8)	-	62.8(8.8)	62.9(7.4)	-
	Mean CompletedModules (sd)	7.1(3.1)	7.3(3.1)	7.2(3.2)	6.8(2.6)	6.4(3.4)	6.8(3.6)	6.5(3.3)	-	4.7(3.7)	6.3(3.0)	-
PanicDisorder	N (%)	475(100)	339(71.4)	80(16.8)	8(1.7)	5(1.1)	18(3.8)	11(2.3)	0(0.0)	10(2.1)	4(0.8)	0(0.0)
	Women (%)	299(62.9)	221(65.2)	54(67.5)	4(50.0)	0.0(0.0)	4(22.2)	11(100.0)	0.0(0.0)	1(10.0)	4(100.0)	0.0(0.0)
	Mean age (sd)	34.8(10.6)	35.6(10.1)	32.7(11.2)	31.8(12.3)	30.0(12.1)	34.9(13.3)	34.3(12.4)	-	31.7(11.8)	28.3(6.4)	-
	Mean InitialGAF^a^ (sd)	60.7(9.5)	61.1(9.3)	58.7(12.2)	60.4(7.4)	61.3(1.5)	60.2(6.7)	58.8(5.5)	-	61.2(5.2)	66.0(12.2)	-
	Mean Completedmodules (sd)	7.1(2.9)	7.4(2.8)	6.8(2.9)	5.8(3.9)	4.0(3.7)	6.3(3.0)	6.6(3.9)	-	6.5(3.3)	5.3(3.7)	-
SocialAnxietydisorder	N (%)	344(100)	222(64.5)	81(23.5)	8(2.3)	4(1.2)	12(3.5)	2(0.6)	0(0.0)	3(0.9)	10(2.9)	2(0.6)
	Women (%)	194(56.4)	130(58.6)	49(60.5)	4(50.0)	1(25.0)	1(8.3)	2(100.0)	0.0(0.0)	0.0(0.0)	7(70.0)	0.0(0.0)
	Mean age (sd)	32.7(10.8)	34.3(11.8)	31.4(8.8)	27.6(3.2)	35.5(7.1)	26.0(6.2)	32.5(12.0)	-	24.7(3.2)	24.6(4.5)	24.0(2.8)
	Mean InitialGAF^a^ (sd)	63.1(7.4)	63.3(7.5)	63.2(7.6)	61.0(4.4)	66.3(5.1)	61.7(3.4)	75.0(−)	-	60.0(0.0)	58.3(8.0)	60.0(0.0)
	Mean CompletedModules (sd)^b^	6.2(3.1)	6.6(3.0)	6.1(3.0)	5.0(3.4)	2.9(1.3)	3.4(2.1)	5.4(6.5)	-	3.4(1.4)	5.0(3.1)	2.7(0.9)

a For the total population, GAF values are based on n = 1327; for Depression, n = 634, for Panic disorder, n = 403, and for Social anxiety disorder n = 290.

b The number of modules completed for patients in social anxiety disorder treatment was rescaled from 12 or 15 treatment modules to a range of 0–10 modules for comparison purposes within the social anxiety disorder group, as well as between diagnostic groups. Ten treatment modules were offered to all patients treated for depression and panic disorder.

### Substance use prevalence

Among the population of 1581 patients, problematic substance use was reported by 32.4%, with 24.1% indicating problematic alcohol use only, 4.6% reporting problematic drug use only and 3.7% indicating combined problematic alcohol and drug use, with a total of 8.3% indicating problematic drug use with or without alcohol. The age range was 17 to 79. Patients treated for depression were slightly older than the population mean, whereas panic disorder and social anxiety disorder patients were younger. The mean GAF-score indicated moderate to mild symptoms of difficulty in social, occupational or school functioning. A higher proportion of women than men were receiving ICBT treatment, particularly for depression and panic disorder. More men than women had harmful alcohol use, probable alcohol dependence and hazardous drug use as well as hazardous drug use combined with problematic alcohol use as measured with AUDIT and DUDIT respectively. In contrast, for harmful drug use the proportion of women was larger as well as for harmful drug use in combination with problematic alcohol use.

### Number of treatment modules completed

The number of treatment modules completed was analyzed by gender, age and diagnostic category across mutually exclusive categories of substance use: non-problematic use, problematic alcohol use, problematic drug use and combined drug and problematic alcohol use. See Table 2.

**Table 2:** Number of completed treatment modules^a^ by gender, age, diagnosis and problematic substance use category.

		Non-problematicusers (n = 1069)	Problematic alcoholusers (n = 381)	Problematic drugusers (n = 72)	Combinedproblematic alcoholand drug users(n = 59)			
		Mean (SD)	Mean (SD)	Mean (SD)	Mean (SD)	*F*	df	*p*
**Gender**	Men (n = 583)	6.6(3.2)	6.2(3.1)	5.2(3.1)	5.2(3.3)	4.1	3,579	0.007
	Women(n = 998)	7.4(2.9)	7.0(3.1)	6.7(3.6)	5.8(3.1)	4.1	3,994	0.007
**Age**	18–24(n = 228)	6.2(3.2)	6.1(3.2)	4.2(3.0)	5.4(3.4)	1.9	3,224	0.129
	25–34(n = 599)	7.1(3.1)	6.7(3.0)	5.1(3.2)	5.6(3.2)	4.9	3,595	0.002
	35–64(n = 720)	7.5(2.9)	6.9(3.2)	7.6(3.1)	5.4(3.2)	3.8	3,716	0.010
	65+ (n = 34)	7.9(2.9)	7.3(3.3)	6.0(−)	-(−)	0.3	2,31	0.774
**Diagnosis**	Depression(n = 762)	7.3(3.1)	7.1(3.1)	6.6(3.4)	5.8(3.3)	2.5	3,758	0.057
	Panicdisorder(n = 475)	7.4(2.8)	6.6(3.0)	6.4(3.3)	6.1(3.3)	2.9	3,471	0.034
	Socialanxietydisorder(n = 344)	6.6(3.0)	5.9(3.0)	3.7(2.7)	4.3(2.7)	7.1	3,340	<0.001

a. Ten treatment modules were offered to all patients treated for depression and panic disorder. The number of modules completed for patients in social anxiety disorder treatment was rescaled from 12 or 15 treatment modules to a range of 0–10 modules for comparison purposes within the social anxiety disorder group and between diagnostic groups.

Tukey-Kramer post-hoc tests showed that men with problematic drug use completed, on average, 1.4 fewer treatment modules than men without problematic substance use (*p = *0.049). Among women, only those with combined problematic drug and alcohol use completed significantly fewer treatment modules (1.6 fewer modules) compared to patients without problematic substance use (*p = *0.018).

Among 25 to 34-year-olds, those with problematic drug use completed an average of 1.94 fewer treatment modules than those without problematic substance use (*p = *0.008). In the group of 35 to 64-year-olds, those with combined problematic alcohol and drug use completed, on average, 2.08 fewer modules than those without problematic substance use (*p = *0.036).

In the group treated for social anxiety disorder, an average of 2.96 fewer treatment modules were completed by patients with problematic drug use (*p = *0.002), as well as 2.30 fewer treatment modules among those with combined problematic alcohol and drug use (*p = *0.023), compared to patients without problematic substance use. In the group treated for panic disorder, which showed significantly fewer completed treatment modules for problematic substance users overall, the post-hoc tests did not show any significant differences.

### Therapy outcomes

A general overview of therapy outcomes for the total population by diagnostic category across mutually exclusive substance use categories is shown in Table 3. Within-group effect sizes (Cohen’s *d*) show effects over time for each problematic use category, and B-values based on ANCOVA analyses, controlled for MADRS-S, PDSS-SR and LSAS-SR baseline values, show mean differences in therapy outcome between problematic users in different categories, compared to non-users who serve as the reference category throughout.

**Table 3:** Therapy outcomes among ICBT patients by treatment group and problematic substance use categories.

		NoProblematicUse	Alcohol Use Categories			Drug Use Categories			Drug Use Categories and Problematic Alcohol Use		
			Hazardous	Harmful	ProbableDependence	Hazardous	Harmful	ProbableDependence	Hazardous	Harmful	ProbableDependence
Depressionn = 762		508	160	24	11	14	15	0	10	20	0
	MADRS-pre,mean (SD)	24.2(6.5)	25.5(6.6)	25.8(6.9)	29.9(6.9)	25.8(5.5)	25.5(5.9)	-	26.3(5.1)	25.5(5.8)	-
	MADRS-post,mean (SD)	13.8(9.0)	14.2(9.2)	16.0(9.1)	18.4(10.5)	15.6(8.7)	17.3(10.7)	-	18.7(10.8)	18.0(8.6)	-
	Within-groupseffect size, *d*	1.33	1.42	1.24	1.36	1.45	0.98	-	0.95	1.05	-
	B^a^		−0.22	1.39	1.66	0.98	2.86	-	3.84	3.52	-
	*p*-value		0.779	0.435	0.524	0.675	0.201	-	0.159	0.071	-
PanicDisordern = 475		339	80	8	5	18	11	0	10	4	0
	PDSS-pre,mean (SD)	12.0(5.5)	12.4(5.3)	9.1(3.8)	16.2(3.4)	12.5(5.8)	14.0(6.2)	-	12.3(5.8)	11.0(3.5)	-
	PDSS-post,mean (SD)	5.6(5.1)	7.0(6.0)	7.3(3.0)	12.4(7.1)	6.3(5.1)	6.0(5.7)	-	6.4(4.2)	5.0(0.0)	-
	Within-groupseffect size, *d*	1.21	0.96	0.56	0.76	1.17	1.41	-	1.24	2.80	-
	B^a^		1.29	2.73	5.29	0.58	−0.30	-	0.97	−0.21	-
	*p*-value		0.034	0.121	0.017	0.626	0.840	-	0.557	0.942	-
SocialAnxietyDisordern = 344		222	81	8	4	12	2	0	3	10	2
	LSAS-pre,mean (SD)	75.9(25.5)	70.0(21.6)	76.9(22.0)	81.0(17.6)	78.7(25.0)	49.0(7.1)	-	84.0(31.2)	82.4(24.1)	91.5(14.9)
	LSAS-post,mean (SD)	57.7(27.2)	54.9(24.5)	71.9(29.5)	55.3(34.7)	77.8(22.5)	41.5(17.7)	-	42.0(21.6)	63.0(23.6)	111.0(12.7)
	Within-groupseffect size, *d*	0.69	0.66	0.21	1.08	0.04	0.79	-	1.92	0.86	−1.99
	B^a^		1.52	13.44	−6.21	18.00	3.51	-	−21.66	0.51	41.84
	*p*-value		0.550	0.056	0.527	0.002	0.800	-	0.056	0.935	0.003

a. B-values describe mean differences in specific outcomes between groups based on ANCOVA analyses, where patients with no problematic substance use serve as the reference category.

Overall within-group effect sizes for MADRS-S were high. While effect sizes were generally high for patients treated for depression, they were nominally lower among those who had concurrent harmful drug use as well as hazardous drug use combined with problematic alcohol use. In contrast, higher within-group effect sizes occurred among hazardous alcohol users as well as hazardous drug users who were treated for depression Among patients treated for panic disorder, effect sizes according to the PDSS-SR ranged between fair and very high. Patients with harmful alcohol use and those with probable dependence on alcohol showed lower effect sizes. Again in contrast, higher effect sizes were identified in the panic disorder group for harmful drug users and those combining harmful drug use with problematic alcohol use. Effect sizes for patients treated for social anxiety disorder ranged from below zero to very high on the LSAS-SR, where patients with harmful alcohol use and hazardous drug use had lower effect sizes and the two patients who showed probable drug dependence in combination with problematic alcohol use had negative effect sizes. In the social anxiety disorder group, higher effect sizes were found for those with either probable dependence on alcohol or combined hazardous drug use with problematic alcohol use.

The ANCOVA analyses showed that individuals with harmful drug use combined with problematic alcohol use negatively affected overall depression outcomes in the entire study population, with an average of 3.5 points post-treatment higher scores on the MADRS-S (not shown). However, among patients treated for depression, substance use did not affect improvement in depressive symptoms. In contrast, panic disorder patients with hazardous use of alcohol (1.29 points higher on the PDSS-SR) or probable dependence (5.29 points higher on the PDSS-SR) and social anxiety disorder patients with hazardous drug use (18 points higher on the LSAS-SR and 5.03 points higher on the MADRS-S) had worse therapy outcomes than non-using patients within their respective diagnostic categories. The two individuals with probable dependence on drugs combined with problematic alcohol use in the social anxiety disorder groups differed highly significantly from the non-users in the same diagnostic category.

### Effects of problematic use on therapy outcomes, controlling for gender, age, baseline GAF and screening scores and number of completed modules

Separate ANCOVA analyses were conducted with therapy outcome as the dependent variable, controlling for age, gender, initial GAF score, baseline MADRS-S score, problematic use, treatment group and number of modules completed. Due to missing data on GAF, the number of individuals in the sample was 1316.

For the group treated for depression, problematic alcohol and drug use did not affect MADRS-S outcomes. However, GAF (B = −0.14, p = 0.003), baseline MADRS-S scores (B = 0.42, p<0.001) and number of completed modules (B = −1.14, p<0.001) significantly affected MADRS-S outcomes in the depression group. For the group treated for panic disorder, controlling for PDSS-SR baseline values and analyzing PDSS-SR outcomes, GAF (B = −0.08, p = 0.001), baseline PDSS-SR scores (B = 0.32, p<0.001) and number of completed modules (B = −0.59, p<0.001) significantly affected outcomes, and individuals with problematic alcohol use (B = 1.55, p = 0.008) had worse outcomes than those without problematic substance use. An in-depth analysis of the substance use subcategories in the panic disorder group showed that patients with exclusive hazardous alcohol use (B = 1.29, p = 0.035) and patients with exclusive probable alcohol dependence (B = 6.35, p = 0.005) had worse outcomes on PDSS-SR than patients without problematic substance use. For the social anxiety disorder group, GAF (B = −0.36, p = 0.046), baseline LSAS-SR scores (B = 0.67, p<0.001) and the number of modules completed (B = −1.48, p<0.001) affected outcomes and individuals with problematic drug use had significantly worse outcomes than patients in this group without problematic substance use (B = 12.431, p = 0.053). An in-depth analysis of the substance use subcategories in the social anxiety disorder group showed that patients with exclusive hazardous drug use (B = 13.70, p = 0.039), patients with combined problematic alcohol use and hazardous drug use (B = −47.62, p<0.001) as well as patients with combined problematic alcohol use and probable drug dependence (B = 35.80, p = 0.008) had worse outcomes on LSAS-SR than patients without problematic substance use.

## Discussion

Generally, according to GAF scores, patients in this study consisted of fairly highly functioning individuals, regardless of substance use severity. The overall prevalence of problematic substance use in the patient population was 32%, with 28% problematic alcohol use and 8% problematic drug use, levels higher than in previous studies of patients in psychiatry [3], [4] as well as the general population [5]. A larger proportion of patients overall were women, but we found more men in the categories of more severe alcohol use and hazardous drug use. Our finding that severe alcohol use was more prevalent among men than among women is consistent with earlier studies [5], [45]. The total prevalence of problematic substance use by diagnostic group was lowest among patients treated for panic disorder (29%), followed by depression (33.4%) and social anxiety disorder (35.5%).

Treatment outcomes for the total population in terms of depression ratings were good, with large effect sizes (Cohen’s *d* 0.81 to 1.09) regardless of problematic use. This implies that concurrent problematic substance use is not an obstacle to obtaining good effects from ICBT treatment, with a clear exception in the two individuals in the social anxiety disorder group who had combined problematic alcohol use and probable drug dependence. In terms of specific diagnoses, treatment outcomes for depression were not affected by problematic substance use. However, among patients treated for panic disorder, hazardous drug use (80 individuals) and probable alcohol dependence (5 individuals) did negatively affect specific outcomes. For social anxiety disorder, the 12 individuals with hazardous drug use and the two with probable drug dependence combined with problematic alcohol use had worse specific treatment outcomes. It is worth noting that some substance use categories in each treatment goup seemed to fare better in terms of within-group effect sizes than non-problematic users. In the social anxiety disorder group, those with probable alcohol dependence and hazardous drug use combined with problematic alcohol use did better than the non-problematic users. These particular findings would be in conflict with the theoretical view that alcohol and drug use function as safety behaviors for persons dealing with social anxiety, serving to protect the individual from dreaded exposure to anxiety. Not experiencing the feared situation in a sober state could hinder patients from disconfirming erroneous beliefs about the consequences of anxiety in social situations, therefore hampering therapeutic progress [46].

In the depression group, better effect sizes occurred for hazardous alcohol *or* hazardous drug users, but not for those with combined use. Finally, in the panic disorder group patients with probable dependence on alcohol or combined hazardous drug use with problematic alcohol use did better in therapy than non-problematic users. The cause of the improved therapeutic outcomes in these groups is not clear from our data, and further investigation into when substance use and therapy have a synergistic effect, and the possible mechanisms behind this could be warranted. What is clear is that these results are well in line with the argument that ICBT seems to have good results in presence of problematic substance use for these groups.

Problematic substance use negatively affected the number of completed treatment modules to a certain extent, with some gender and age effects. Problematic substance use seems not to have affected the number of modules completed in patients under 25 years of age. Among those older, there were effects of either problematic drug use alone (25–34 years) or the combination of problematic alcohol and drug use (35–64 years). Interpreting data on the number of completed treatment modules presents some challenges. In the ICBT unit’s experience, completing more treatment modules is associated with better outcomes, but an exact defining point regarding the need for individuals to complete a certain number of modules is elusive: some patients benefit the most early on and subsequently lessen their activity in the program without prejudicing good outcomes. Thus there is great variability between individuals regarding the effects of quitting treatment early on therapy outcomes. Nonetheless, the significant overall association between number of modules completed and positive outcome justifies the ambition of ensuring a high rate of completed modules among patients.

### Strengths and limitations

To our knowledge this is the first study of substance use in a population receiving psychotherapeutic treatment over the Internet in regular care. Major strengths of this study are that the data were collected in a non-experimental naturalistic clinical setting, and the sizes of the treatment groups studied were large with nearly complete data sets. These factors suggest high external validity for our findings. Also, the study used well validated measures of substance use and symptom severity.

At the same time, the retrospective design led to several limitations that should be noted. First, our analyses were limited to data that had already been collected at the clinic, thus omitting potentially vital data for this type of study. For instance, no data were collected on the types of drugs used by patients. We were thus unable to establish any connection between type of drug use and therapy outcomes. A substance that is of relevance to the study, but was not addressed in the substance use screening, is nicotine. There is a known connection between nicotine and psychiatric comorbidity [47], particularly affective disorders [48], [49]. Nicotine use would therefore constitute a valuable observation in relation to patient outcomes. Second, the data did not include any information on simultaneous medication parallel to ICBT treatment, nor on access to other treatment providers. What we did know was that psychiatric medication needed to be stable for one month prior to beginning ICBT and that patients receiving concurrent psychotherapy were not eligible for ICBT. Simultaneous complementary (e.g., substance use) or alternative treatment access could significantly affect patient outcomes, and lack of this information could bias our conclusions. Third, no data were available on individuals who were referred for ICBT treatment but never began therapy.

An additional limitation is that clinical exclusion of patients with severe substance use may have affected population characteristics and led to results biassed for better functioning patients with less severe substance use problems. At the same time, in this study we were limited to using self-reported data on substance use, and research has shown that self-report data may be subject to bias in form of underreporting actual use [50], [51], so consumption levels may have been higher than we knew. Indeed, if the self-reported substance use in this study was underreported by the patients, this would lend further strength to the conclusion that ICBT is effective even at slightly higher levels of problematic use. Of course, this reasoning is only speculative. Nonetheless, some studies have shown that respondents report higher consumption of substances when filling out self-report instruments on a computer in comparison to paper questionnaires, so actual use may have been reported relatively correctly [52], [53]. In any case, in the absence of corroborative measures of substance use, for example biological markers, we were not able to evaluate the validity of the level of substance use reported by patients. A further limitation concerned the classification of patients in categories of mutually exclusive types of substance use, which in some cases led to very small groups. It is unclear whether these would be representative of future patient populations, even if the our patients did constitute the entire population of patients at the ICBT unit during the time period studied. A final limitation is the fact that the substance use categories defined were based on baseline measures, since the naturalistic study design did not include follow-up measures of substance use. This makes it impossible to know whether changes in initial substance use categories occurred during treatment and how this in turn might have affected treatment outcome.

### Future research

Future research should use a prospective design, where important data that were missing in this study could be included. Information on types of substances and concurrent medication used by the participating parties would make for analyses providing more information on the actual effects on therapy and also a more detailed prevalence description. A more in-depth study of substance use and therapy progress during the actual treatment period could investigate why certain user categories seem to do better in therapy than their non-problematic counterparts. Data on reasons for exclusion gathered by admitting psychiatrists would further add knowledge about the patient group studied and add information on generalizability to a psychiatric population.

## Conclusion

The results indicate that problematic substance use generally does not affect the outcome of ICBT. In most cases, problematic substance use had no discernible detrimental effect on outcomes, suggesting no obstacle to providing individuals with ICBT based on problematic substance use. It should be noted, however, that particular subgroups of individuals in specific categories of substance use did differ in outcome from their counterparts without problematic substance use. At the same time, increased severity of substance use neither worsened nor improved outcomes; neither did it reduce the number of treatment modules completed in any linear correlation. This made it difficult to evaluate the exact effects of substance use on outcomes and treatment completion.

Notwithstanding the above, we conclude that the worse outcomes and fewer treatment modules completed among men and younger adult patients (25–34 years old) with problematic drug use, as well as women and older patients (35–64 years old) with combined problematic drug and alcohol use, do give cause for further attention to these groups. These individuals’ substance use might need to be addressed concurrently with treatment for their primary psychiatric diagnosis.

## References

[1] Regier DA, Farmer ME, Rae DS, Locke BZ, Keith SJ, et al (1990) Comorbidity of mental-disorders with alcohol and other drug-abuse-results from the epidemiologic catchment-area (ECA) study. J Am Med Assoc 264: 2511–2518. 2232018

[2] Grant BF, Stinson FS, Dawson DA, Chou SP, Dufour MC, et al (2004) Prevalence and co-occurrence of substance use disorders and independent mood and anxiety disorders-Results from the national epidemiologic survey on alcohol and related conditions. Arch Gen Psychiatry 61: 807–816. 15289279 10.1001/archpsyc.61.8.807

[3] Eberhard S, Nordstrom G, Hoglund P, Ojehagen A (2009) Secondary prevention of hazardous alcohol consumption in psychiatric out-patients: a randomised controlled study. Soc Psychiatry Psychiatr Epidemiol 44: 1013–1021. 19294323 10.1007/s00127-009-0023-7

[4] Cruce G, Nordström L, Öjehagen A (2007) Risky use and misuse of alcohol, drugs and cigarettes detected by screening questionnaires in a clinical psychosis unit. Nord J Psychiat 61: 92–99. 10.1080/08039480701226062 17454723

[5] Sinadinovic K, Wennberg P, Berman AH (2011) Population screening of risky alcohol and drug use via Internet and Interactive Voice Response (IVR): A feasibility and psychometric study in a random sample. Drug Alcohol Depend 114: 55–60. 21030164 10.1016/j.drugalcdep.2010.09.004

[6] Khantzian EJ (1985) The self-medication hypothesis of addicctive disorders-focus on heroin and cocaine dependence Am J Psychiatry. 142: 1259–1264. 10.1176/ajp.142.11.1259 3904487

[7] Bolton JM, Robinson J, Sareen J (2009) Self-medication of mood disorders with alcohol and drugs in the National Epidemiologic Survey on Alcohol and Related Conditions. J Affect Disord 115: 367–375. 19004504 10.1016/j.jad.2008.10.003

[8] Bolton JM, Cox B, Clara I, Sareen J (2006) Use of alcohol and drugs to self-medicate anxiety disorders in a nationally representative sample. J Nerv Ment Dis 194: 818–825. 17102705 10.1097/01.nmd.0000244481.63148.98

[9] Buckner JD, Timpano KR, Zvolensky MJ, Sachs-Ericsson N, Schmidt NB (2008) Implications of comorbid alcohol dependence among individuals with social anxiety disorder. Depress Anxiety 25: 1028–1037. 18781667 10.1002/da.20442 PMC2778209

[10] Zimmermann P, Wittchen HU, Höfler M, Pfister H, Kessler RC, et al (2003) Primary anxiety disorders and the development of subsequent alcohol use disorders: A 4-year community study of adolescents and young adults. Psychol Med 33: 1211–1222. 14580076 10.1017/s0033291703008158

[11] Schneier FR, Foose TE, Hasin DS, Heimberg RG, Liu SM, et al (2010) Social anxiety disorder and alcohol use disorder co-morbidity in the national epidemiologic survey on alcohol and related conditions. Psychol Med 40: 977–988. 20441690 10.1017/S0033291709991231 PMC2917264

[12] Kenneson A, Funderburk JS, Maisto SA (2013) Substance use disorders increase the odds of subsequent mood disorders. Drug Alcohol Depend 133: 338–343. 23906994 10.1016/j.drugalcdep.2013.06.011

[13] Fergusson DM, Boden JM, Horwood LJ (2009) Tests of Causal Links Between Alcohol Abuse or Dependence and Major Depression. Arch Gen Psychiatry 66: 260–266. 19255375 10.1001/archgenpsychiatry.2008.543

[14] Kushner MG, Abrams K, Borchardt C (2000) The relationship between anxiety disorders and alcohol use disorders: A review of major perspectives and findings. Clin Psychol Rev 20: 149–171. 10721495 10.1016/s0272-7358(99)00027-6

[15] Bruce SE, Yonkers KA, Otto MW, Eisen JL, Weisberg RB, et al (2005) Influence of psychiatric comorbidity on recovery and recurrence in generalized anxiety disorder, social phobia, and panic disorder: A 12-year prospective study. Am J Psychiatry 162: 1179–1187. 15930067 10.1176/appi.ajp.162.6.1179 PMC3272761

[16] McEvoy PM, Shand F (2008) The effect of comorbid substance use disorders on treatment outcome for anxiety disorders. J Anxiety Disord 22: 1087–1098. 18164585 10.1016/j.janxdis.2007.11.007

[17] Eskildsen A, Hougaard E, Rosenberg NK (2010) Pre-treatment patient variables as predictors of drop-out and treatment outcome in cognitive behavioural therapy for social phobia: A systematic review. Nord J Psychiat 64: 94–105. 10.3109/08039480903426929 20055730

[18] Andersson G, Cuijpers P, Carlbring P, Lindefors N (2007) Effects of Internet-delivered cognitive behaviour therapy for anxiety and mood disorders. Review Series: Psychiatry 2: 9–14.

[19] Cuijpers P, Donker T, van Straten A, Li J, Andersson G (2010) Is guided self-help as effective as face-to-face psychotherapy for depression and anxiety disorders? A systematic review and meta-analysis of comparative outcome studies. Psychol Med 40: 1943–1957. 20406528 10.1017/S0033291710000772

[20] Riper H, Andersson G, Christensen H, Cuijpers P, Lange A, et al.. (2010) Theme Issue on E-Mental Health: A Growing Field in Internet Research. J Med Internet Res 12. 10.2196/jmir.1713 PMC3057318 21169177

[21] Titov N (2011) Internet-delivered psychotherapy for depression in adults. Curr Opin Psychiatry 24: 18–23. 20827199 10.1097/YCO.0b013e32833ed18f

[22] Gainsbury S, Blaszczynski A (2011) A systematic review of Internet-based therapy for the treatment of addictions. Clin Psychol Rev 31: 490–498. 21146272 10.1016/j.cpr.2010.11.007

[23] Mueser KT, Noordsy DL, Drake RE, Fox L (2003) Integrated Treatment for Dual Disorders: A Guide to Effective Practice. New York: The Guilford Press.

[24] Hedman E, Ljótsson B, Kaldo V, Hesser H, El Alaoui S, et al (2014) Effectiveness of Internet-based cognitive behaviour therapy for depression in routine psychiatric care. J Affect Disord 155: 49–58. 24238951 10.1016/j.jad.2013.10.023

[25] Hedman E, Ljótsson B, Rück C, Bergström J, Andersson G, et al (2013) Effectiveness of Internet-based cognitive behaviour therapy for panic disorder in routine psychiatric care. Acta Psychiatr Scand 128: 457–467. 23406572 10.1111/acps.12079

[26] Sheehan DV, Lecrubier Y, Sheehan KH, Amorim P, Janavs J, et al (1998) The Mini-International Neuropsychiatric Interview (MINI): The development and validation of a structured diagnostic psychiatric interview for DSM-IV and ICD-10. J Clin Psychiatry 59: 22–33. 9881538

[27] Saunders JB, Aasland OG, Babor TF, Delafuente JR, Grant M (1993) Development of the alcohol-use disorders identification test (AUDIT) - who collaborative project on early detection of persons with harmful alcohol-consumption-II. Addiction 88: 791–804. 8329970 10.1111/j.1360-0443.1993.tb02093.x

[28] Bergman H, Kallmen H (2002) Alcohol use among Swedes and a psychometric evaluation of the Alcohol Use Disorders Identification Test. Alcohol Alcohol 37: 245–251. 12003912 10.1093/alcalc/37.3.245

[29] Sinadinovic K, Berman AH, Hasson D, Wennberg P (2010) Internet-based assessment and self-monitoring of problematic alcohol and drug use. Addict Behav 35: 464–470. 20092953 10.1016/j.addbeh.2009.12.021

[30] Babor TF, Higgins-Biddle JC, Saunders JB, Monteiro MG (2001) AUDIT The Alcohol Use Disorders Identification Test: Guidelines for Use in Primary Care. Geneva: World Health Organization.

[31] Berman AH, Bergman H, Palmstierna T, Schlyter F (2005) Evaluation of the Drug Use Disorders Identification Test (DUDIT) in criminal justice and detoxification settings and in a Swedish population sample. Eur Addict Res 11: 22–31. 15608468 10.1159/000081413

[32] American Psychiatric Association (1987) Diagnostic and Statistical Manual of mental Disorders, Third edition, Revised. Washington, DC: American Psychiatric Association.

[33] Jones SH, Thornicroft G, Coffey M, Dunn G (1995) A brief mental-health outcome scale-reliability and validity of the global assessment of functioning (GAF). Br J Psychiatry 166: 654–659. 7620753 10.1192/bjp.166.5.654

[34] Svanborg P, Asberg M (1994) A new self-rating scale for depression and anxiety-states based on the comprehensive psychopathological rating-scale. Acta Psychiatr Scand 89: 21–28. 8140903 10.1111/j.1600-0447.1994.tb01480.x

[35] Montgomery SA, Asberg M (1979) New depression scale designed to be sensitive to change. Br J Psychiatry 134: 382–389. 444788 10.1192/bjp.134.4.382

[36] Svanborg P, Ekselius L (2003) Self-assessment of DSM-IV criteria for major depression in psychiatric out- and inpatients. Nord J Psychiat 57: 291–296. 10.1080/08039480307281 12888397

[37] Carlbring P, Brunt S, Bohman S, Austin D, Richards J, et al (2007) Internet vs. paper and pencil administration of questionnaires commonly used in panic/agoraphobia research. Comput Human Behav 23: 1421–1434.

[38] Houck PR, Spiegel DA, Shear MK, Rucci P (2002) Reliability of the self-report version of the Panic Disorder Severity Scale. Depress Anxiety 15: 183–185. 12112724 10.1002/da.10049

[39] Shear MK, Brown TA, Barlow DH, Money R, Sholomskas DE, et al (1997) Multicenter collaborative panic disorder severity scale. Am J Psychiatry 154: 1571–1575. 9356566 10.1176/ajp.154.11.1571

[40] Furukawa TA, Shear MK, Barlow DH, Gorman JM, Woods SW, et al (2009) Evidence-Based Guidelines for Interpretation of the Panic Disorder Severity Scale. Depress Anxiety 26: 922–929. 19006198 10.1002/da.20532 PMC2760657

[41] Hedman E, Ljotsson B, Ruck C, Furmark T, Carlbring P, et al (2010) Internet administration of self-report measures commonly used in research on social anxiety disorder: A psychometric evaluation. Comput Human Behav 26: 736–740.

[42] Liebowitz MR (1987) Social phobia. Mod Probl Pharmacopsychiatry 22: 141–173. 2885745 10.1159/000414022

[43] Mennin DS, Fresco DM, Heimberg RG, Schneier FR, Davies SO, et al (2002) Screening for social anxiety disorder in the clinical setting: using the Liebowitz Social Anxiety Scale. J Anxiety Disord 16: 661–673. 12405524 10.1016/s0887-6185(02)00134-2

[44] Howell DC (2002) Statistical methods for psychology. Pacific Grove, CA: Duxbury/Thomson Learning.

[45] Wilsnack RW, Wilsnack SC, Kristjanson AF, Vogeltanz-Holm ND, Gmel G (2009) Gender and alcohol consumption: patterns from the multinational GENACIS project. Addiction 104: 1487–1500. 19686518 10.1111/j.1360-0443.2009.02696.x PMC2844334

[46] Clark DM, Wells A (1995) A cognitive model of social phobia. In: Heimberg RG, Hope D, Schneier FR, editors. Social phobia: diagnosis, assessment, and treatment. New York: Guildford Press. 69–93.

[47] John U, Meyer C, Rumpf H-J, Hapke U (2004) Smoking, nicotine dependence and psychiatric comorbidity–a population-based study including smoking cessation after three years. Drug Alcohol Depend 76: 287–295. 15561479 10.1016/j.drugalcdep.2004.06.004

[48] Murphy JM, Horton NJ, Monson RR, Laird NM, Sobol AM, et al (2003) Cigarette smoking in relation to depression: Historical trends from the Stirling County Study. Am J Psychiatry 160: 1663–1669. 12944343 10.1176/appi.ajp.160.9.1663

[49] Boden JM, Fergusson DM, Norwood LJ (2010) Cigarette smoking and depression: tests of causal linkages using a longitudinal birth cohort. Br J Psychiatry 196: 440–446. 20513853 10.1192/bjp.bp.109.065912

[50] Harrison LD, Hughes A (1997) The validity of self-reported drug use: Improving the accuracy of survey estimates Rockville, MD: US Department of Health and Human Services, National Institutes of Health.

[51] Stockwell T, Donath S, Cooper-Stanbury M, Chikritzhs T, Catalano P, et al (2004) Under-reporting of alcohol consumption in household surveys: a comparison of quantity-frequency, graduated-frequency and recent recall. Addiction 99: 1024–1033. 15265099 10.1111/j.1360-0443.2004.00815.x

[52] Kallmen H, Sinadinovic K, Berman AH, Wennberg P (2011) Risky drinking of alcohol in Sweden: A randomized population survey comparing web- and paper-based self-reports. Nord Stud Alcohol Drugs 28: 123–130.

[53] Wang Y-C, Lee C-M, Lew-Ting C-Y, Hsiao CK, Chen D-R, et al (2005) Survey of substance use among high school students in Taipei: Web-based questionnaire versus paper-and-pencil questionnaire. J Adolesc Health 37: 289–295. 16182139 10.1016/j.jadohealth.2005.03.017

